# *Cutibacterium acnes* Phylotype I and II Strains Interact Differently With Human Skin Cells

**DOI:** 10.3389/fcimb.2020.575164

**Published:** 2020-11-16

**Authors:** Karl-Jan Spittaels, Ruben Ongena, Christos C. Zouboulis, Aurélie Crabbé, Tom Coenye

**Affiliations:** ^1^ Laboratory of Pharmaceutical Microbiology, Ghent University, Ghent, Belgium; ^2^ Departments of Dermatology, Venereology, Allergology and Immunology, Dessau Medical Center, Brandenburg Medical School Theodor Fontane, Dessau, Germany

**Keywords:** acne vulgaris, *Cutibacterium acnes*, *Propionibacterium acnes*, keratinocytes, sebocytes, bacterial association, epithelial barrier, invasion

## Abstract

Acne vulgaris is one of the most common skin disorders and affects the pilosebaceous units. Although the exact pathogenesis of acne is still unknown, *Cutibacterium acnes* (formerly known as *Propionibacterium acnes*) is considered one of the key contributing factors. In fact, a significant association exists between *C. acnes* strains belonging to phylotype I and acne. However, there is still heavy debate on the exact role of *C. acnes* in acne and its behavior in the pilosebaceous unit, and more specifically its interactions with the human skin cells. In this study, key elements of the host-pathogen interaction were studied for a collection of *C. acnes* strains, belonging to phylotype I and II, including association with HaCaT keratinocytes and SZ95 sebocytes, the effect of *C. acnes* on keratinocyte tight junctions in a HaCaT monoculture and in an additional keratinocyte-sebocyte co-culture model, and *C. acnes* invasion through the keratinocyte cell layer. Our data showed association of all *C. acnes* strains to both skin cell lines, with a significantly higher association of type I strains compared to type II strains. Microscopic imaging and western blot analysis of the tight junction protein ZO-1, together with transepithelial electrical resistance (TEER) measurements revealed an initial induction of keratinocyte tight junctions after 24 h infection but a degradation after 48 h, demonstrating a decline in cell lining integrity during infection. Subsequently, *C. acnes* was able to invade after 48 h of infection, although invasion frequency was significantly higher for type II strains compared to type I strains.

## Introduction

Acne vulgaris is one of the most common skin disorders with a prevalence ranging from 35% to nearly 100% in teenagers and young adults, depending on the country and specific age group (Moradi Tuchayi et al., 2015; Heng and Chew, 2020). Despite its common occurrence, the etiology of acne is still not completely clear, although there is a consensus that the origin of this skin disorder is multifactorial. Acne is a disease of the pilosebaceous unit, and its hallmarks include dysfunctional keratinocyte differentiation, overproduction of sebum by sebocytes and alterations in its lipid fractions, *C. acnes* colonization, and a marked inflammatory response (Moradi Tuchayi et al., 2015; Dréno, 2017; Hazarika, 2019). In addition, differences in the composition of the skin microbiome, particularly in the relative composition of the *Cutibacterium acnes* populations, seem to correlate with acne presentation (Barnard et al., 2016; Dagnelie et al., 2018).

*C. acnes* (formerly known as *Propionibacterium acnes*) (Scholz and Kilian, 2016) is a Gram-positive, anaerobic but aerotolerant bacterium, and a member of the human skin microbiota. It thrives in lipophilic environments and is mostly found on skin regions that possess the highest amount of pilosebaceous units (including shoulders, neck, face, and chest) (Gribbon et al., 1993; Grice et al., 2009) where it can be present in high numbers (up to 10^6^ CFU/cm²) (Leyden et al., 1998).

Based on cell wall sugar analysis and serological agglutination tests, *C. acnes* was first divided into two distinct serotypes (Johnson and Cummins, 1972). Screening of the sequences of nine housekeeping genes, including the *recA* and *tly* genes, later found that *C. acnes* can be grouped into three major phylotypes (McDowell et al., 2005; Lomholt and Kilian, 2010). This resulted in the division of *C. acnes* into three subspecies: *Cutibacterium acnes* subspecies *acnes* (type I), *C. acnes* subsp*. defendens* (type II) (McDowell et al., 2016), and *C. acnes* subsp*. elongatum* (type III) (Dekio et al., 2015). Recent research has suggested that type I strains are more often associated with acne vulgaris, while type II strains are more frequently found on healthy skin or in deep tissue infections (Lomholt and Kilian, 2010; McDowell, 2017).

*C. acnes* is typically located inside the pilosebaceous unit, where it forms microcolonies and biofilms (Jahns et al., 2012; Jahns and Alexeyev, 2014). The presence of such a bacterial biofilm, combined with hyperkeratinization and excess sebum production, results in blocking of the pilosebaceous unit, leading to the formation of comedones (Cunliffe et al., 2000; Gollnick, 2015). Subsequent production of host tissue degrading enzymes, including lipases and proteases, and virulence factors like the CAMP factor (Nakatsuji et al., 2011; Spittaels and Coenye, 2018) may lead to weakening of the integrity of the cell lining of the pilosebaceous unit, ultimately leading to invasion of *C. acnes* into the dermis, inflammation, and the development of acne (Beylot et al., 2014).

While the presence of *C. acnes* in the pathology of acne has been well established, little is known about the interaction between *C. acnes* and the epidermal cells and whether there are differences between strains that are typically associated with acne (type I) or healthy skin (type II). Indeed, previous studies have mostly emphasized the immunostimulatory effect of this bacterium or its virulence factors on human cells (O’Neill and Gallo, 2018). Furthermore, most *in vitro* models used lack the multicellular complexity of the *in vivo* parental tissue (Kanwar et al., 2018). We studied the capacity of *C. acnes* to associate with keratinocyte and sebocyte monolayers, as association is the initial step in biofilm formation, as well as invasion in host cells and tissue. A large panel of *C. acnes* strains, belonging to both type I and II, was included in this study. Next, the integrity of keratinocyte tight junctions in the presence of *C. acnes* was investigated. Additionally, a physiologically relevant co-culture model was developed in which keratinocytes and sebocytes are co-cultivated to study infection with *C. acnes*. In this model, the effect of *C. acnes* on the tight junction integrity was confirmed and bacterial invasion was monitored over time.

## Materials and Methods

### Bacteria and Cell Cultures

Fifteen *C. acnes* strains belonging to phylotype I and phylotype II were obtained from BEI Resources, NIAID, NIH as part of the Human Microbiome Project (Manassas, VA, United States), while the LMG16711 strain was obtained from the BCCM/LMG Bacteria Collection (Ghent, Belgium) (**Table 1**). The bacteria were cultured on reinforced clostridium agar (RCA; LabM, Heywood, UK) for 3 days at 37°C under anaerobic conditions [Anaerogen Compact system (Oxoid, Aalst-Erembodegem, Belgium) or Gaspak EZ system (BD, VWR, Leuven, Belgium)].

**Table 1:** *C. acnes* strains used.

Strain	Biological origin	*recA* type	MLST type^a^
**LMG16711^T^**	Human acneic skin	IA	IA_1_
**HL001PA1**	Human normal skin	II	II
**HL002PA1**	Human acneic skin	IB	IA_2_
**HL027PA1**	Human normal skin	IB	IA_2_
**HL043PA2**	Human acneic skin	IA	IA_1_
**HL045PA1**	Human acneic skin	IA	IA_1_
**HL050PA2**	Human normal skin	II	II
**HL053PA1**	Human acneic skin	IA	IA_1_
**HL056PA1**	Human normal skin	IA	IA_1_
**HL059PA1**	Human normal skin	IB	IA_2_
**HL059PA2**	Human normal skin	IB	IA_2_
**HL060PA1**	Human acneic skin	II	II
**HL072PA1**	Human acneic skin	IA	IA_1_
**HL082PA2**	Human acneic skin	II	II
**HL110PA3**	Human acneic skin	II	II
**HL110PA4**	Human acneic skin	II	II

^a^According to eMLST by McDowell et al. (2012).

The immortalized human sebaceous gland cell line SZ95 (Zouboulis et al., 1999) was cultivated in Sebomed basal medium (Biochrom, Berlin, Germany) supplemented with 10% fetal bovine serum (FBS; Gibco, Life Technologies Corporation, NY, United States), 1% penicillin/streptomycin (pen/strep; 100 UI/ml, Sigma-Aldrich, Steinheim, Germany), 5 ng/ml human epidermal growth factor (Thermo-Fisher, MA, United States), and 1 mM CaCl_2_.

HaCaT cells (spontaneously immortalized human keratinocytes) (Boukamp et al., 1988) were cultivated in Dulbecco’s modified Eagle medium (DMEM; Gibco, Life Technologies Corporation, NY, United States) supplemented with 10% FBS and 1% pen/strep. For experiments in which the function of the tight junctions of the HaCaT cells was investigated, the cells were cultivated for 5 days in DMEM after which the medium was changed to the supplemented Sebomed medium for an additional 2 days. This medium contains a high calcium concentration which is needed for keratinocyte differentiation and the assembly of tight junctions (Micallef et al., 2009; Elsholz et al., 2014). Both cell lines were maintained at 37°C in a humidified atmosphere containing 5% CO_2_.

### Stimulation of Human Skin Cells With Live and Heat-Killed Bacteria, and Bacterial Association

The HaCaT and SZ95 cells were seeded in 24-well cell culture plates (Greiner Bio-One, Frickenhausen, Germany) at a density of 2.5 x 10^4^ cells per well and incubated until confluency was reached after 7 days. Fresh medium containing pen/strep was added every 2 days, except in the final change in which medium without antimicrobials was used. *C. acnes* strains, grown anaerobically for 24 h in Sebomed basal medium were centrifuged for 5 min at 3500 rpm (Eppendorf centrifuge 5804 R, Eppendorf, Hamburg, Germany) and the bacterial pellets washed with phosphate buffered saline (PBS; Gibco, Life Technologies Corporation). These bacterial pellets were resuspended in PBS and used to infect the wells containing the SZ95 or HaCaT cells with a multiplicity of infection of 10:1. The infected cells were incubated anaerobically using the Anaerogen Compact system. After 48 h infection under anaerobic conditions at 37°C, the supernatant was removed and the wells were washed with PBS to remove non-associated bacteria. Finally, 1 mL 0.1% Triton X-100 (Sigma Aldrich) in PBS was added to the wells and the content was vigorously pipetted to detach the cells and associated bacteria. The number of host cell associated bacteria was then determined after plating on RCA. The number of *C. acnes* in the supernatant, i.e. not associated with the skin cells, was also determined by plating on RCA. To obtain dead bacteria, suspensions of one type I strain (HL053PA1) and one type II strain (HL110PA3) in PBS were heated for 30 min at 75°C. HaCaT cells were exposed to the heat-killed bacteria with numbers corresponding to MOI of 10:1 and 100:1.

### Modified Gram Stain

To image bacterial association to the human skin cells, a modified Gram stain was used based on a previously developed protocol (Becerra et al., 2016). Cells were infected as described above, followed by anaerobic incubation at 37°C. After 48 h, the supernatant was removed, the wells washed twice with PBS and 750 µL 4% paraformaldehyde (PFA; Electron Microscopy Sciences, Hatfield, England) in PBS was added to fixate the cells. After 20 min of fixation, the PFA solution was removed, the wells were washed twice with PBS and the modified Gram stain was applied. Briefly, 500 µL of a 0.5% crystal violet solution (Pro-Lab Diagnostics, Bromborough, England) was added to the wells for 5 min, after which the wells were rinsed twice with 1 mL H_2_O to remove excess crystal violet. Then, 500 µL Gram’s iodine (Pro-Lab Diagnostics) was added for 1 min and again rinsed with 1 mL H_2_O. Next, 1 mL denatured alcohol (ethanol with 1% isopropyl alcohol and 1% butanone; Chem-Lab NV, Zedelgem, Belgium) was added for 30–60 s, before quickly rinsing with 1 mL H_2_O. Then, 500 µL 0.25% safranin (Pro-Lab Diagnostics) was added for 1 min and the wells were rinsed again with 1 mL H_2_O. This normal Gram stain procedure was followed by two extra dehydration steps using 1 mL 95% and 1 mL 100% ethanol (Sigma Aldrich) and ending with a 10 min stain using 750 µL 6% alcoholic saffron (VWR, Leuven, Belgium). Finally, the wells were rinsed with 1 mL H_2_O and 1 mL PBS was added during microscopic analysis using an EVOS FL Auto Imaging System (Life technologies, Ca, United States) equipped with a 20x objective (final magnification: 368x).

### Immunocytochemistry and Fluorescence Microscopy

The HaCaT cells were infected with *C. acnes* and fixed as described above. The immunocytochemical staining of ZO-1 was performed as follows: non-specific binding sites were blocked using blocking buffer containing 8% bovine serum albumin (BSA; Sigma Aldrich) and 0.5% Triton X-100 in PBS for 1 h. Next, the sample was incubated for 1 h with a 1:50 dilution of human ZO-1 antibody (Invitrogen, Fisher Scientific) in PBS and washed 3 times with a 0.5% Tween 20 (Sigma Aldrich) solution in PBS. A 1:500 secondary antibody (Goat anti-Mouse IgG, Alexa Fluor 488; Invitrogen, Fisher Scientific) dilution in blocking buffer was added during 1 h and the samples were again washed with PBS. All samples were incubated at room temperature and protected from light. Finally, a droplet of DAPI (Invitrogen, Fisher Scientific) was added to stain the cell nuclei and the samples were visualized using the EVOS FL Auto Imaging System fluorescence microscope equipped with a 20x objective (Life Technologies) (final magnification: 599x). Confocal microscopy was performed by the Centre for Advanced Light Microscopy at Ghent University (Belgium). The images were recorded on a Nikon C2 confocal laser scanning module attached to a motorized Nikon Ti2-E inverted microscope (Nikon Benelux) equipped with a 60×/1.4 Plan Apo VC Oil immersion objective (CFI Plan Apo VC, Nikon). A 488 nm continuous wave laser (Coherent Sapphire) was used for excitation. Images were recorded by unidirectional scanning without averaging, with a pinhole size of 30 µm. A Z-step of 0.5 µm and a pixel size of 100 nm was used.

### Protein Extraction and Western Immunoblotting

Proteins were extracted from the infected HaCaT cells using Pierce RIPA buffer (Thermo-Fisher) supplemented with protease and phosphatase inhibitors (Sigma Aldrich). 100 µL of this extraction buffer was used to lyse the cells while scraping the bottom of the well for full lysis. The content of two wells was pooled, transferred to an Eppendorf tube and incubated on ice for 10 min. The cell lysate was then centrifuged at 14,000 rpm for 10 min at 4°C and the supernatant subsequently transferred to a new Eppendorf tube, diluted with Laemmli buffer (Bio-Rad, CA, United States) and boiled for 10 min. Afterwards, the protein extracts were size-separated on 10% SDS-PAGE gels and subsequently transferred to nitrocellulose membranes. The membranes were blocked using 5% BSA in PBS, and incubated overnight with the primary antibodies at 4°C. Specific mouse antibodies were used to detect ZO-1 (diluted 1:500; Invitrogen, Fisher Scientific) and β-actin (diluted 1:1000; LifeSpan biosciences, Bioconnect). The membranes were subsequently washed and incubated for 2 h with the anti-mouse IgG horseradish peroxidase-conjugated secondary antibody (diluted 1:10,000; Invitrogen, Fisher Scientific). Then, the blots were developed and the protein bands visualized using the ChemiDoc Imaging system (Bio-Rad). Intensity of Western blot bands was quantified by densitometry using the ImageJ software and normalization of the ZO-1 band intensity by using β-actin.

### Development of a Co-Culture Model of Keratinocytes and Sebocytes

SZ95 cells were seeded in a 24-well plate in supplemented Sebomed basal medium as described above, while HaCaT cells were seeded at a density of 2.5 x 10^4^ cells per insert (ThinCert membrane pore diameter 8 µm, Greiner Bio-One) with supplemented DMEM. Both cell lines were incubated separately for 5 days after which the medium of both cultures was removed and the wells or inserts were washed with PBS. The inserts containing the HaCaT cells were then transferred to the 24-well cell culture plate containing SZ95 cells and supplemented Sebomed medium without pen/strep was added to the wells and the inserts. After 2 days of incubation, the inserts were infected with *C. acnes* at an MOI of 10:1 as described above and incubated anaerobically using the Anaerogen Compact system. In this model, there is direct contact between *C. acnes* and the keratinocytes, and indirect contact with the sebocytes, similar to the *in vivo* pilosebaceous unit (**Figure 5A**).

### Transepithelial Electrical Resistance Measurement

Transepithelial electrical resistance (TEER) of the epithelial barrier formed by the HaCaT keratinocytes was measured using an epithelial voltmeter connected to a pair of STX2 chopstick electrodes (EVOM^2^, World Precision Instruments, Florida, United States).

### Host Cell Viability

Viability of the HaCaT and SZ95 cell cultures was determined using a lactate dehydrogenase activity assay kit (LDH assay; Sigma Aldrich) according to the manufacturer’s instructions.

### Invasion Assay

*C. acnes* invasion was studied using the developed co-culture model of HaCaT and SZ95 cells. Invasion of *C. acnes* through the HaCaT cell layer in the insert was investigated by infecting the co-culture model as described above. The amount of *C. acnes* present in the underlying wells was determined by plating 100 µL samples, taken from the wells after 24, 48, and 72 h infection, on RCA.

### Statistics

All experiments contain a minimum of three biological replicates and were analyzed using SPSS Statistics version 25. Two groups were compared using One-Sample or Independent-Samples t-Tests or Mann-Whitney U-tests depending on the normality of the data sets. For experiments comparing more than two groups, a one-way analysis of variance (ANOVA) or a Kruskal-Wallis test was performed. The data were considered statistically significant at a p-value ≤ 0.05. All data are expressed as means ± standard error of the means (SEM).

## Results

### Viability of Keratinocytes and Sebocytes After Infection With Different Amounts of *C. acnes*

First, viability of the SZ95 sebocytes and HaCaT keratinocytes grown as monolayer cells in the presence of *C. acnes* was assessed at different multiplicities of infection (MOI), using lactate dehydrogenase (LDH) activity as a measure for cytotoxicity. A type I (LMG16711) and type II (HL060PA1) strain were tested at an MOI of 1:1, 10:1, and 100:1 during 24 and 48 h of anaerobic infection. No significant increase in LDH activity was observed for all tested MOIs compared to the uninfected controls up to 48 h (**Supplementary Data S1**). An MOI of 10:1 was used for further experiments.

### Type I *C. acnes* Strains Associate More With Human Skin Cells Than Type II Strains

**Figure 1:**
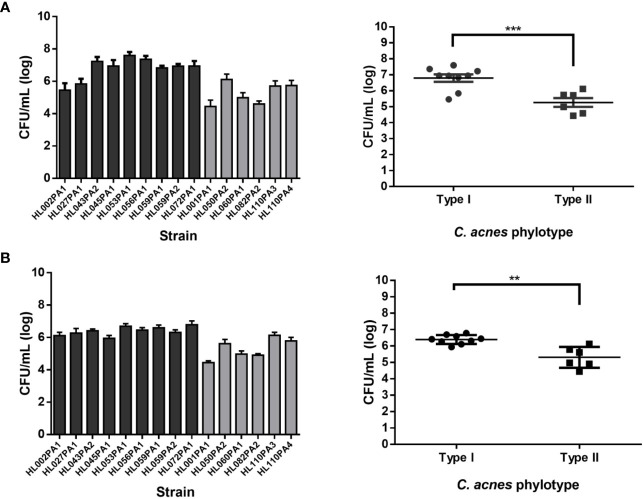
Left graphs show results per strain (black bars: phylotype I, grey bars: phylotype II) while right graphs show results grouped per phylotype. **(A)** Bacterial association with SZ95 sebocytes expressed as CFU/mL (log scale). **(B)** Bacterial association with HaCaT keratinocytes expressed as CFU/mL (log scale). Data shown are mean from at least five biological replicates, error bars indicate SEM. **p < 0.01, ***p < 0.005.

**Figure 2:**
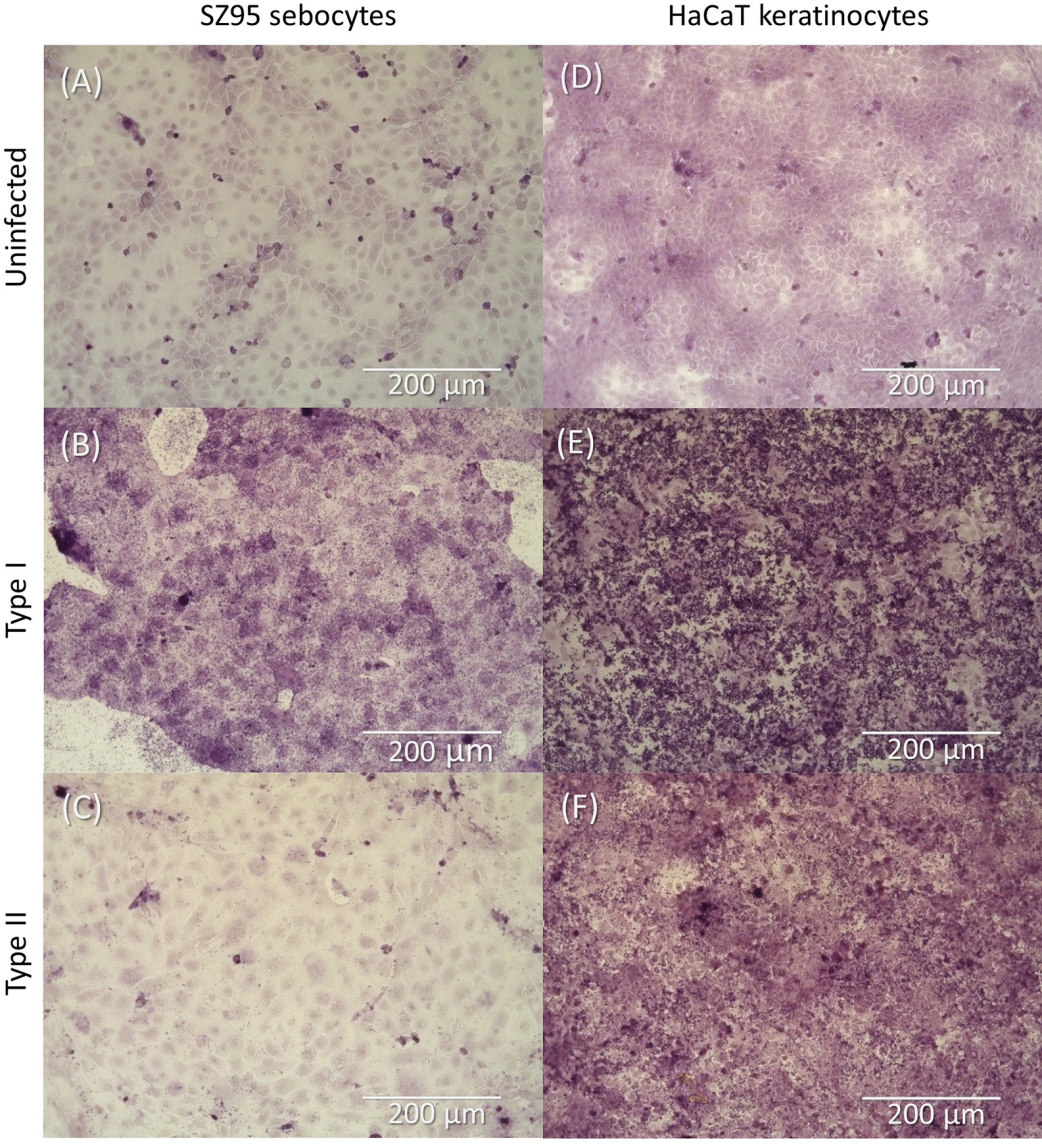
Light microscopy image of SZ95 sebocytes **(A–C)** and HaCaT keratinocytes **(D–F)** after infection with *C. acnes* and a modified Gram stain. **(A)** Uninfected SZ95 cells, **(B)** SZ95 cells infected with HL053PA1 (type I strain), and **(C)** SZ95 cells infected with HL110PA3 (type II strain). **(D)** Uninfected HaCaT cells, **(E)** HaCaT cells infected with HL053PA1, and **(F)** HaCaT cells infected with HL110PA3. Total magnification: 368x. Scale bars: 200 µm.

Fifteen *C. acnes* strains were tested for association with human SZ95 sebocyte and HaCaT keratinocyte cells (grown as confluent monolayers in a 24-well cell culture plate). All *C. acnes* strains tested associated with both skin cell lines (**Figure 1**). Type I strains showed significantly higher association (6.96 ± 0.17 log CFU/mL) with SZ95 cells than type II strains (5.26 ± 0.31 log CFU/mL) (p=0.0011). Similar results were obtained with HaCaT cells, to which type I strains also associate significantly more than type II strains (6.40 ± 0.09 log CFU/mL and 5.31 ± 0.26 log CFU/mL respectively, p=0.007). These differences were confirmed using light microscopy of samples after a modified Gram stain (**Figure 2**). The number of non-associated bacteria in the supernatant was also determined by plating; these numbers did not differ between type I and II strains (**Supplementary Data S2**).

### *C. acnes* Induces Changes in Cell Lining Integrity of Keratinocytes

**Figure 3:**
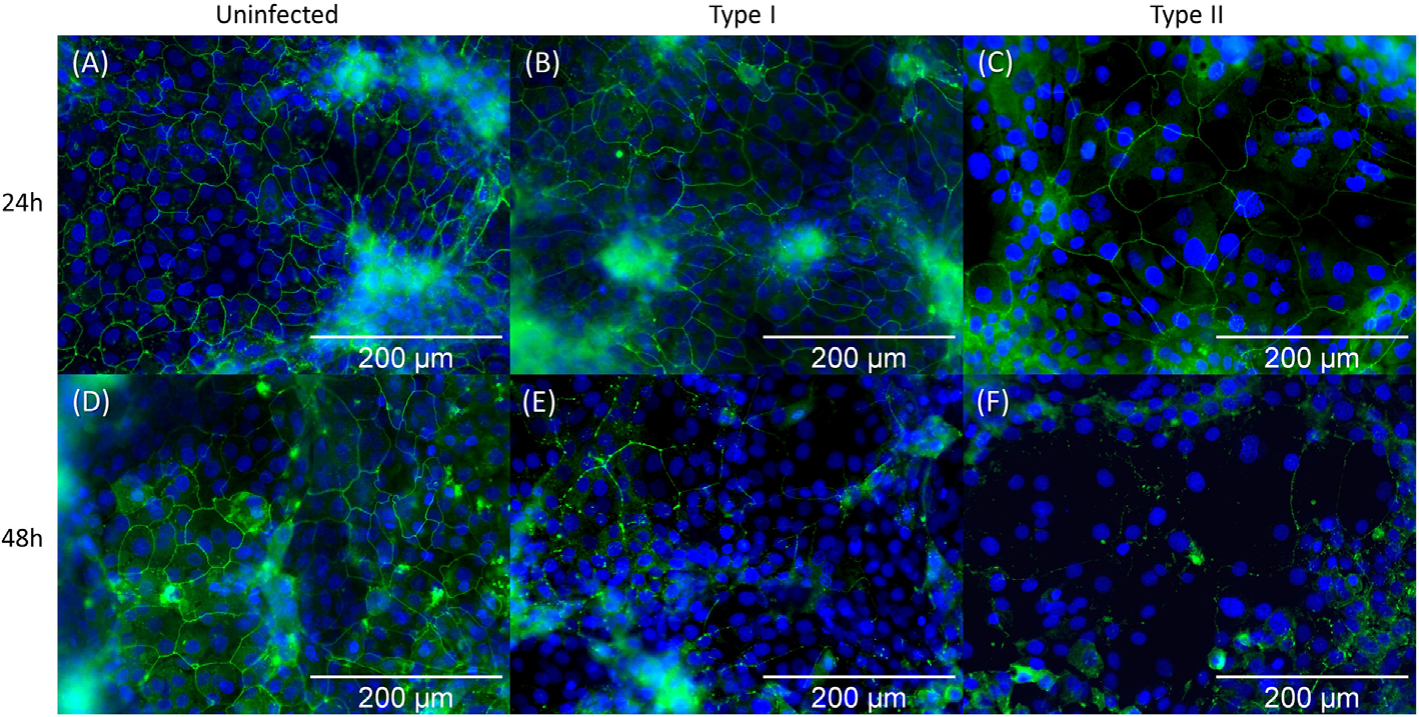
Immunocytochemistry staining of the ZO-1 tight junction protein of HaCaT keratinocytes followed by fluorescence microscopy. ZO-1 tight junction proteins and the cell nuclei (DAPI) are visualized in green and blue respectively. Pictures **(A–C)** represent the state after 24 h infection: **(A)** uninfected control, **(B)** infected with HL053PA1 (type I strain), and **(C)** infected with HL110PA3 (type II strain). Pictures **(D–F)** show the remains of ZO-1 protein after 48 h infection: **(D)** uninfected control, **(E)** infected with HL053PA1, and **(F)** infected with HL110PA3. Total magnification: 599x. Scale bars: 200 µm.

**Figure 4:**
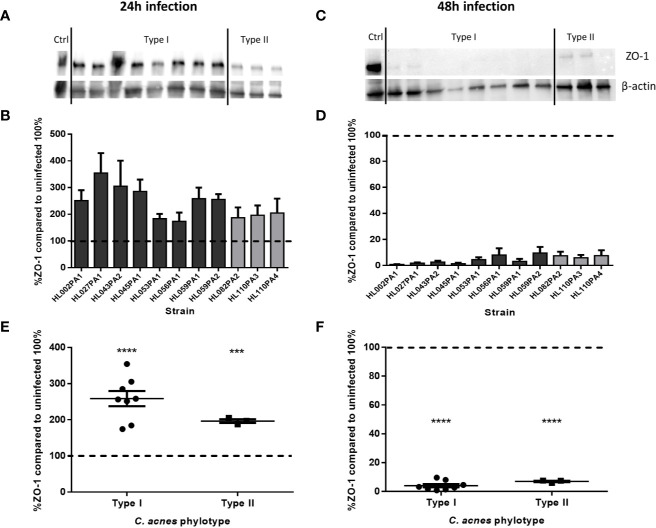
Levels of ZO-1 in infected HaCaT keratinocytes after 24 h **(A, B)** and 48 h **(C, D)**. When data are analyzed per phylotype, there is no difference in ZO-1 levels after 24 h **(E)** or after 48 h **(F)**, although there is a significant increase **(E)** and decrease **(F)** after 24 and 48 h respectively compared to the uninfected control (dotted line). Data shown are mean from at least three biological replicates, error bars indicate SEM. ***p < 0.005, ****p < 0.001.

We subsequently investigated the effect of *C. acnes* on the keratinocyte cell lining integrity by imaging the organization of the HaCaT tight junction protein ZO-1 after 24 and 48 h of infection with *C. acnes*. Immunocytochemistry showed no visible differences in ZO-1 between the control and the infected cells after 24 h (**Figure 3**). However, there was a visible difference between the control and infected cells after 48 h, indicating a relocation or degradation of the ZO-1 proteins after 48 h infection with *C. acnes*; this was observed for both phylotypes. Next, western blot analysis was performed to quantify the ZO-1 protein levels (**Figures 4A, C**). Densitometry of the normalized ZO-1 bands revealed an approximately two-fold increase in ZO-1 for all strains tested compared to the uninfected control at the 24 h infection time point, indicating an initial induction of ZO-1 during the infection process (**Figure 4B**). In contrast, the average ZO-1 level decreased to approximately 5% of the uninfected control after 48 h, indicating a nearly complete degradation of this protein (**Figure 4D**). When *C. acnes* strains were grouped per type, a significant increase in ZO-1 was found after 24 h in lysates infected with both type I (p<0.001) and type II strains (p=0.003) compared to the control (**Figure 4E**). After 48 h infection a significant decrease in ZO-1 levels was observed compared to the control for both type I (p<0.001) and type II strains (p<0.001)(**Figure 4F**). No differences in ZO-1 induction or degradation were observed between the two phylotypes. When cells were exposed to heat-killed *C. acnes* for 48 h, no degradation of ZO-1 was observed (**Supplementary Data S3**).

### Establishment of a Physiologically Relevant Keratinocyte-Sebocyte Co-Culture Model and Its Use to Study *C. acnes* Invasion

**Figure 5:**
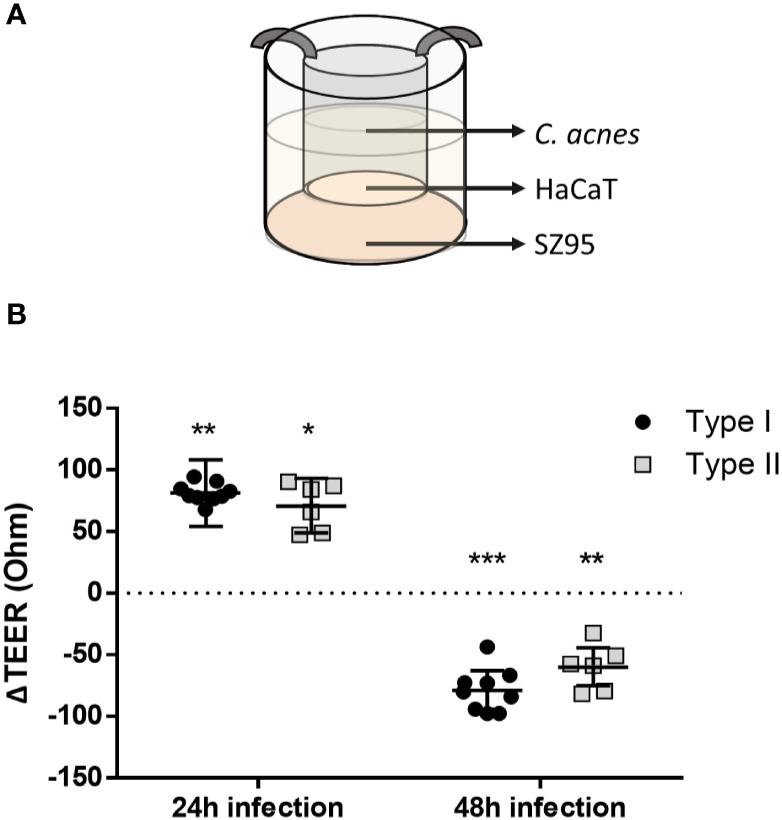
Schematic overview of the co-culture model: SZ95 sebocytes are cultured in a cell culture plate in Sebomed medium for 5 days, HaCaT keratinocytes are cultured in inserts for 5 days in DMEM, after which both are co-cultured for an additional 2 days in Sebomed medium (high calcium concentration), before *C. acnes* is added to the insert (MOI of 10:1) **(A)**. ΔTEER after 24 and 48 h of infection, compared to uninfected control (dotted line) **(B)**. *p < 0.05, **p < 0.01, ***p < 0.005.

**Figure 6:**
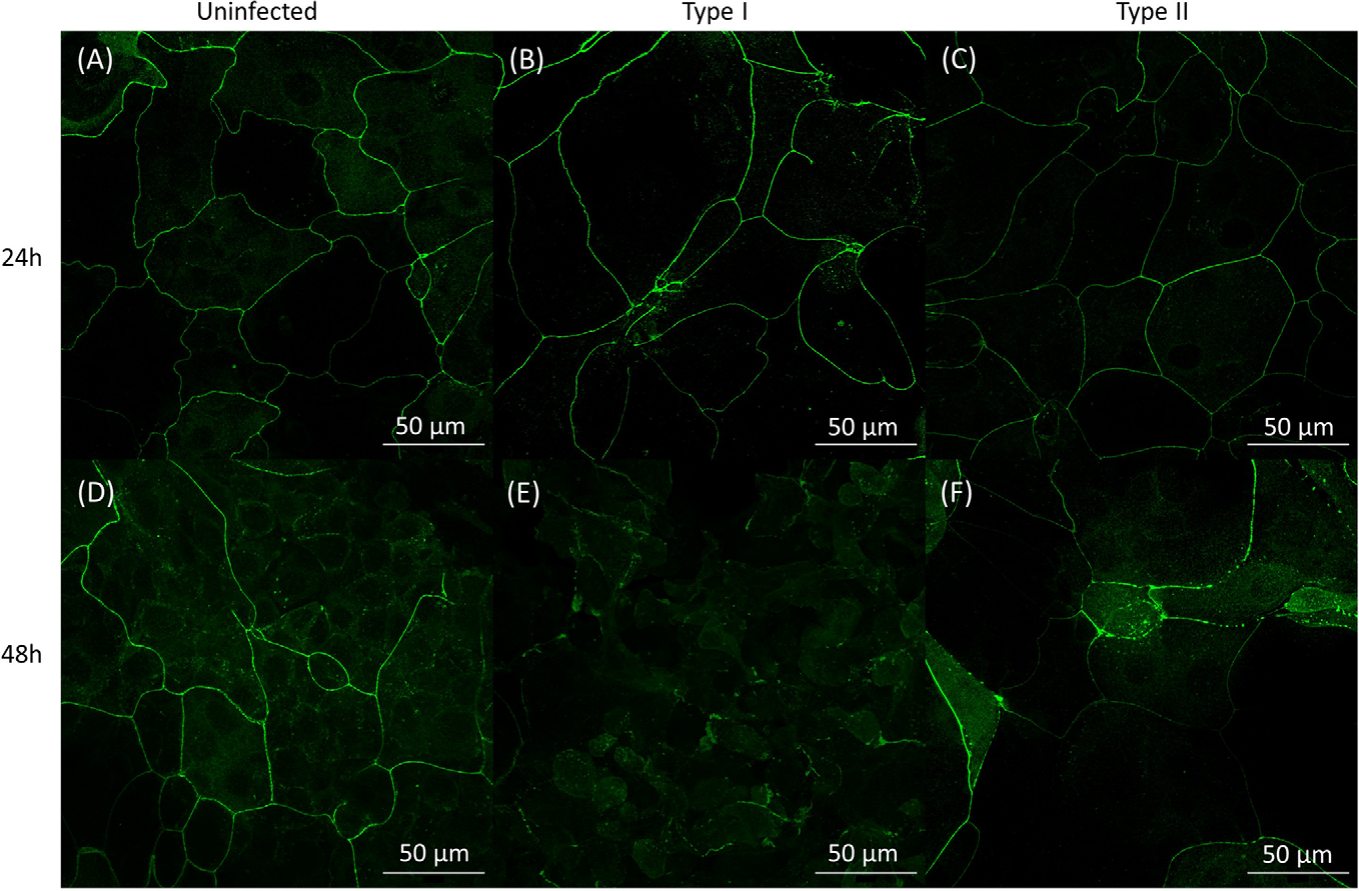
Confocal images of HaCaT keratinocytes grown in the co-culture model labeled with a human ZO-1 antibody (green). Panels **(A–C)**: data obtained after 24 h: **(A)** no infection; **(B)** infection with HL053PA1, a type I strain; **(C)** infection with HL110PA3, a type II strain. Panels **(D–F)**: data obtained after 48 h: **(D)** no infection; **(E)** infection with HL053PA1, a type I strain; **(F)** infection with HL110PA3, a type II strain. Scale bars: 50 µm.

In order to mimic the *in vivo* environment of the pilosebaceous unit more closely, sebocytes and keratinocytes were cultivated together using a cell culture insert (**Figure 5A**). To investigate the integrity of the keratinocyte layer grown on the membrane of the insert, the transepithelial electrical resistance (TEER) was measured before, and after 24 and 48 h of infection. After 24 h the TEER was significantly higher in infected keratinocytes than in the uninfected control (p<0.0099 for type I strains and p=0.0215 for type II strains) (**Figure 5B**). However, after 48 h infection, the TEER was significantly lower in infected inserts for both types compared to the infected control. When exposed to heat-killed *C. acnes*, an increase in TEER was measured in the inserts for both types after 24 h. However, the TEER did not differ significantly from the control after 48 h exposure to heat-killed *C. acnes*. This suggests the need for live bacteria to degrade the cell lining integrity (**Supplementary Data S4**). Furthermore, this disruption of the keratinocyte layer integrity by live bacteria was confirmed by confocal imaging ZO-1 on the cell inserts in the co-culture model (**Figure 6**), showing less ZO-1 at the cell-cell interface.

**Figure 7:**
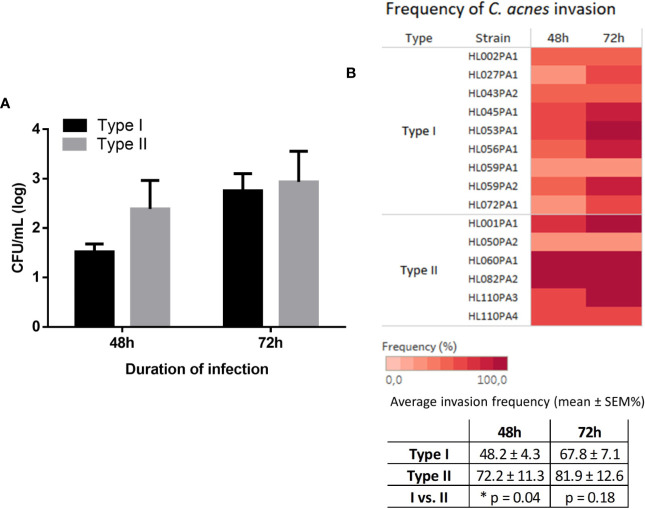
Average CFU/mL (log scale) of *C. acnes* recovered from the wells after 48 and 72 h infection grouped per type **(A)**. The frequency at which the different *C. acnes* strains invaded, i.e. the percentage of times the biological replicates were found in the wells, is illustrated in a heat map and table **(B)**. No invasion was observed after 24 h (not shown). Data were collected from at least four biological replicates, bars represent mean ± SEM.

Finally, we investigated whether *C. acnes* is capable of invading from the insert through the keratinocyte cell layer into the wells of the cell culture plate. Samples were taken from the wells every 24 h for up to 3 days of infection. While no growth was observed in samples taken after 24 h infection, *C. acnes* was found in the wells after 48 and 72 h. Strains from both types were able to invade through the HaCaT cell layer and no significant differences in CFU/mL were found between the two phylotypes (**Figure 7A**). However, the invasion frequency differed between strains, i.e. the % of biological replicates that successfully invaded. On average, for type I strains 48% of the biological replicates were able to invade after 48 h, whereas this was 72% for type II strains (p=0.04) (**Figure 7B**).

### Type I Strains of *C. acnes* Are More Cytotoxic in a Keratinocyte-Sebocyte Co-Culture

**Figure 8:**
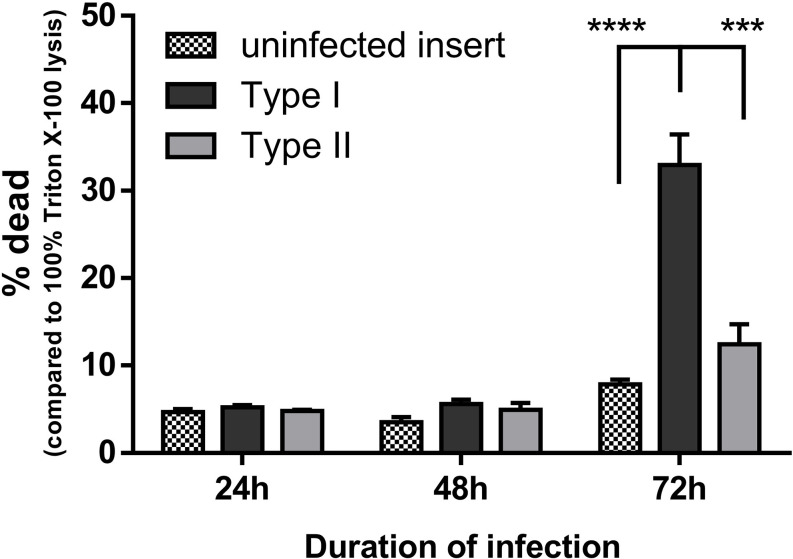
HaCaT cell viability during infection with type I or type II *C. acnes* strains was monitored using LDH activity assays. Cell viability is expressed as the percentage of dead cells compared to 100% dead cells after lysis with 1% Triton X-100 in PBS. Data shown are the mean from at least three biological replicates, error bars indicate SEM. ***p < 0.005, ****p < 0.001.

Cell viability during the infection process in the developed keratinocyte-sebocyte co-culture model was determined using the LDH assay (**Figure 8**). After 24 h of infection the fraction of dead cells was equal in the control group and the inserts infected with type I or II strains. Similar results were obtained after 48 h infection. After 72 h however, the percentage of dead cells increased to over 30% when infected with type I strains, while still being less than 10% in the control group. In contrast, only a slight, but not significant increase in dead cells (approximately 12%) was observed after infection with type II strains.

## Discussion

Altered follicular keratinization, increased and altered sebum production, *C. acnes* colonization, and inflammation are traditionally described as the key factors in the pathogenesis of acne. Nevertheless, the specific role of the skin commensal *C. acnes* in the whole disease process remains unclear (McLaughlin et al., 2019). To this end, we studied the interaction process of *C. acnes* with its host, hereby focusing on potential differential interactions of *C. acnes* strains typically associated with acneic skin (type I) or with healthy skin (type II).

*C. acnes* macrocolonies of more than 1,000 bacterial cells can be observed in association with the follicular epithelium of the pilosebaceous unit; they are more often present in follicles from acneic skin biopsies than in follicles of individuals with healthy skin (Jahns et al., 2012). High bacterial colonization of the follicles was recently also observed in transmission electron microscopy images of microcomedones (Josse et al., 2020). As *C. acnes* growth and proliferation in the pilosebaceous unit is considered one of the factors that contributes to the etiology of acne, we first investigated the ability of strains belonging to phylotypes I and II to associate with HaCaT keratinocytes and SZ95 sebocytes. Our data indicate that strains belonging to both types can associate with keratinocytes and sebocytes, but higher association of type I strains to both cell types was observed. Not much is known about association of *C. acnes* to human skin cells. *C. acnes* cell-cell adherence and clumping is known to be initiated by cutaneous lipids such as triolein and diolein with maximum cell-cell aggregation in the presence of oleic acid, which is a component of human sebum (Gribbon et al., 1993). A comparative proteome analysis of *C. acnes* isolates from different phylotypes revealed differences in the expressed surface proteins, including adhesion proteins, between those phylotypes (Yu et al., 2016). Interestingly, the 58 kDa surface protein DsA1 that binds host cell-surface proteins is produced by most strains belonging to *C. acnes* type I, while type II and III strains do not produce this protein (Wadström and Ljungh, 1999; Grange et al., 2017). Hence, the absence of DsA1 on the cell walls of type II strains might explain the difference we observed in association between the two types.

The observed disruption of tight junctions in HaCaT keratinocytes (grown as single culture monolayers or co-cultured with SZ95 sebocytes) by both *C. acnes* phylotypes after 48 h infection may contribute to the pathogenesis of acne as it may lead to decreased integrity of the epithelial barrier. Disruption of barrier integrity results in enhanced permeability to proinflammatory cytokines, pathogen associated molecular patterns, and even pathogens, which contributes to the disease process (Lee et al., 2018).

Next, the HaCaT epithelial barrier function was investigated in the co-culture model. After 24 h, both live and dead *C. acnes* temporarily induced barrier function in HaCaT cells (as reflected in higher TEER values). Since *C. acnes* is a member of the normal skin microbiota, the observed increase of ZO-1 after 24 h of infection should not come as a surprise. Resident skin microbiota promote skin barrier function and inhibit potential pathogenic bacteria to invade the human body. Resident bacteria such as *Staphylococcus epidermidis* are known to enhance the production of tight junction proteins through the activation of Toll-like receptors (Cogen et al., 2008). An increase in TEER after 24 h could thus be explained by activation of the Toll-like receptors 2 (TLR2) on the keratinocytes by *C. acnes* peptidoglycan (Yuki et al., 2011; Su et al., 2016). Keratinocytes treated with lysates of *Lactobacillus rhamnosus* GG or *Bifidobacterium longum*, showed an increased TEER that peaked after 24 h and an increased expression of the tight junction proteins, including ZO-1. In contrast, inhibition of TLR2 activation using a neutralizing antibody abolished this effect, indicating a correlation between TLR2 recognition and strengthening of the tight junctions (O’Neill et al., 2013). Confocal imaging of the HaCaT cells grown in this co-culture model after immunostaining of ZO-1 as well as the TEER values confirmed degradation of this tight junction protein after 48 h of infection with live *C. acnes*.

After degradation of tight junction proteins, permeability of the epithelial barrier is increased (Kirschner et al., 2013; Bäsler et al., 2017), possibly leading to the penetration and invasion of pathogens (Awad et al., 2017). This is in agreement with our data in the co-culture model, in which invasion of *C. acnes* was only found after 48 h, corresponding to the time point where ZO-1 levels had dropped strongly and the TEER had decreased significantly. Although all strains tested were able to invade through the keratinocyte cell layer, into the wells, the invasion frequency differed. Strains belonging to type II had a higher invasion frequency compared to strains belonging to type I, an observation that could link the higher frequency of deep tissue infections, in for example prostate tissue, to type II *C. acnes* (Mak et al., 2013; Romano-Bertrand et al., 2014). It should be noted that invasion occurred starting at 48 h, during which, according to cell viability assays and microscopy, HaCaT cells were still viable and attached to the insert. Therefore, *C. acnes* was able to invade through a layer of viable keratinocytes.

In the present study, we investigated bacterial association and the potentially invasive characteristics of a wide panel of *C. acnes* strains belonging to phylotype I or II. Our results shed light into possible reasons why type I strains are associated with acne, while strains belonging to type II are associated with healthy skin or deep tissue infections (Lomholt and Kilian, 2010; McDowell et al., 2012; McDowell et al., 2013; Lomholt et al., 2017). Our results demonstrated a significantly higher bacterial association of type I strains with human skin cells compared to type II strains. This difference could subsequently contribute to the differential colonization of human pilosebaceous units by *C. acnes* and the association of type I strains with acne. Interestingly, strains from both phylotypes induced the breakdown of the epithelial barrier after 48 h of infection, in the monolayer and co-culture model. Finally, bacterial invasion was observed starting at 48 h of infection, which could be attributed to the decrease of the epithelial barrier integrity at this time point. Remarkably, bacterial invasion was significantly more frequent for type II strains compared to type I strains, which could explain the association of phylotype II to opportunistic or deep tissue infections (Mak et al., 2013; McDowell et al., 2013). In this study, we have demonstrated differences in bacterial association, invasion, and cell toxicity between type I and II, that can be linked to disease association of these types. However, more research is needed to elucidate the importance of these differences.

## Data Availability Statement

The original contributions presented in the study are included in the article/**Supplementary Materials**. Further inquiries can be directed to the corresponding author.

## Author Contributions

Conception and design of study: K-JS and TC. Acquisition and analysis of data: K-JS and RO. Drafting of article and/or critical revision: K-JS, CZ, RO, AC, and TC. All authors contributed to the article and approved the submitted version.

## Conflict of Interest

The authors declare that the research was conducted in the absence of any commercial or financial relationships that could be construed as a potential conflict of interest.

## Supplementary Material

Supplementary Data Sheet 1 Determination of *C. acnes* MOI in SZ95 **(A)** and HaCaT cells **(B)** after 24 h (black bars) and 48 h (grey bars) of infection. No significant cell toxicity was observed for MOIs reaching 100:1. Data shown are mean from at least three biological replicates, error bars indicate SEM.

Supplementary Data Sheet 2
*C. acnes* counts of the non-associated fraction after 48 h infection with SZ95 **(A)** or HaCaT cells **(B)**. Data shown are mean from at least three biological replicates, error bars indicate SEM.

Supplementary Data Sheet 3 Immunocytochemistry staining of the ZO-1 tight junction protein of HaCaT keratinocytes followed by fluorescence microscopy after exposure to heat-killed *C. acnes* for 24 and 48 h. ZO-1 tight junction proteins and the cell nuclei (DAPI) are visualized in green and blue respectively. Pictures **(A–C)** show ZO-1 after 24 h exposure to heat-killed HL053PA1: **(A)** control, **(B)** exposed to heat-killed *C. acnes* approximating an MOI of 10:1 for 24 h, and **(C)** exposed to heat-killed *C. acnes* approximating an MOI of 100:1 for 24 h. Pictures **(D–F)** show the still intact ZO-1 protein after 48 h exposure to heat-killed *C. acnes*: **(D)** control, **(E)** heat-killed *C. acnes* approximating an MOI of 10:1, and **(F)** heat-killed *C. acnes* approximating an MOI of 100:1. Total magnification: 599x. Scale bars: 200 µm.

Supplementary Data Sheet 4 The effect of heat-killed *C. acnes* on the cell lining integrity as measured by TEER. After 24 h exposure a significant increase can be observed at the highest concentrations of heat-killed bacteria. Data represent mean, error bars indicate SEM. * p<0.05, *** p<0.005.
