# Supplementary figures and images for: Cutibacterium acnes Phylotype I and II Strains Interact Differently With Human Skin Cells
Source: Front Cell Infect Microbiol. 2020 Nov 16;10:575164. doi: 10.3389/fcimb.2020.575164 (PMC7717938; doi:10.3389/fcimb.2020.575164)

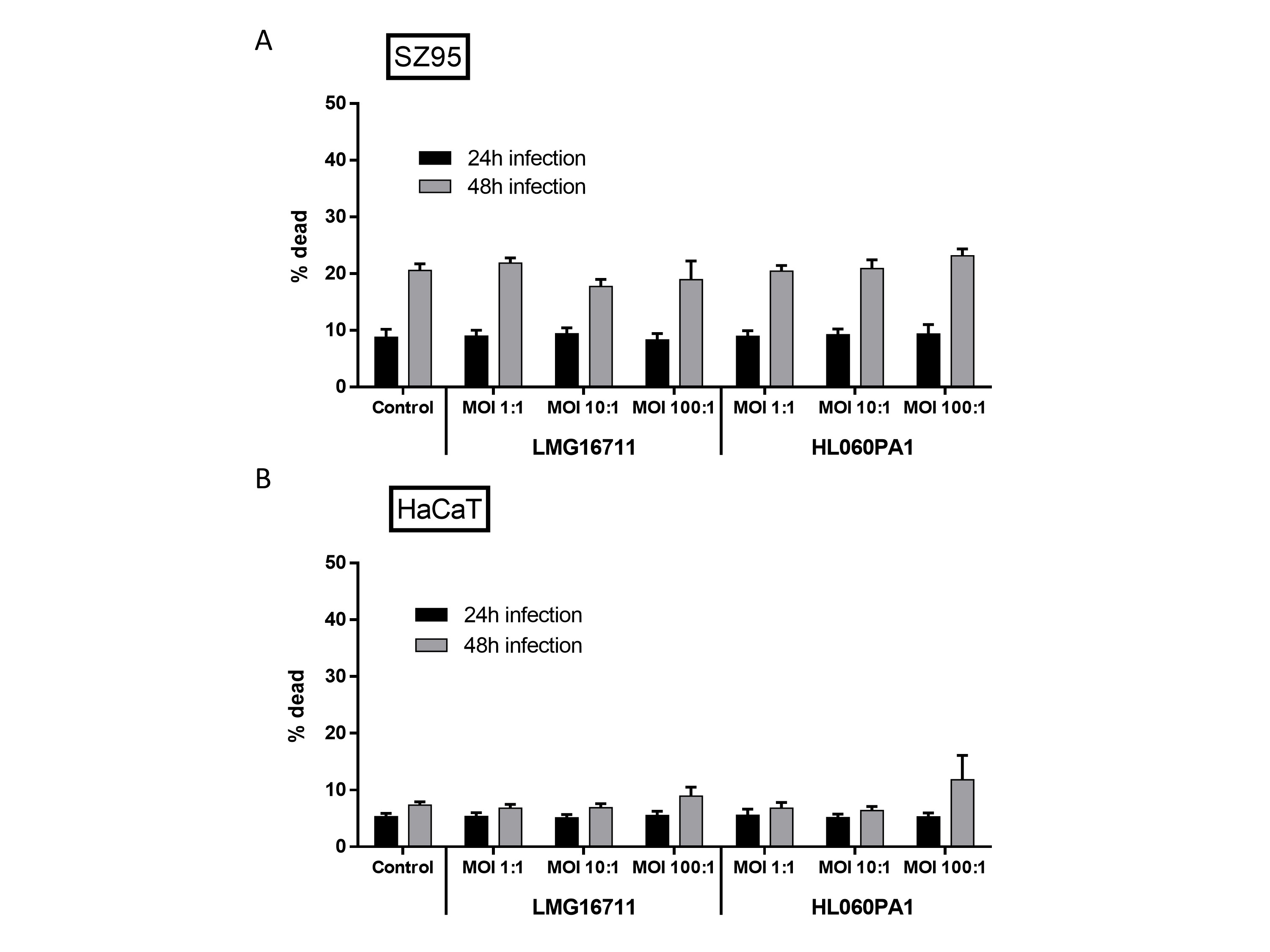

Supplement: Supplementary Data Sheet 1 — Determination of C. acnes MOI in SZ95 (A) and HaCaT cells (B) after 24 h (black bars) and 48 h (grey bars) of infection. No significant cell toxicity was observed for MOIs reaching 100:1. Data shown are mean from at least three biological replicates, error bars indicate SEM. [file Image_1.jpeg]

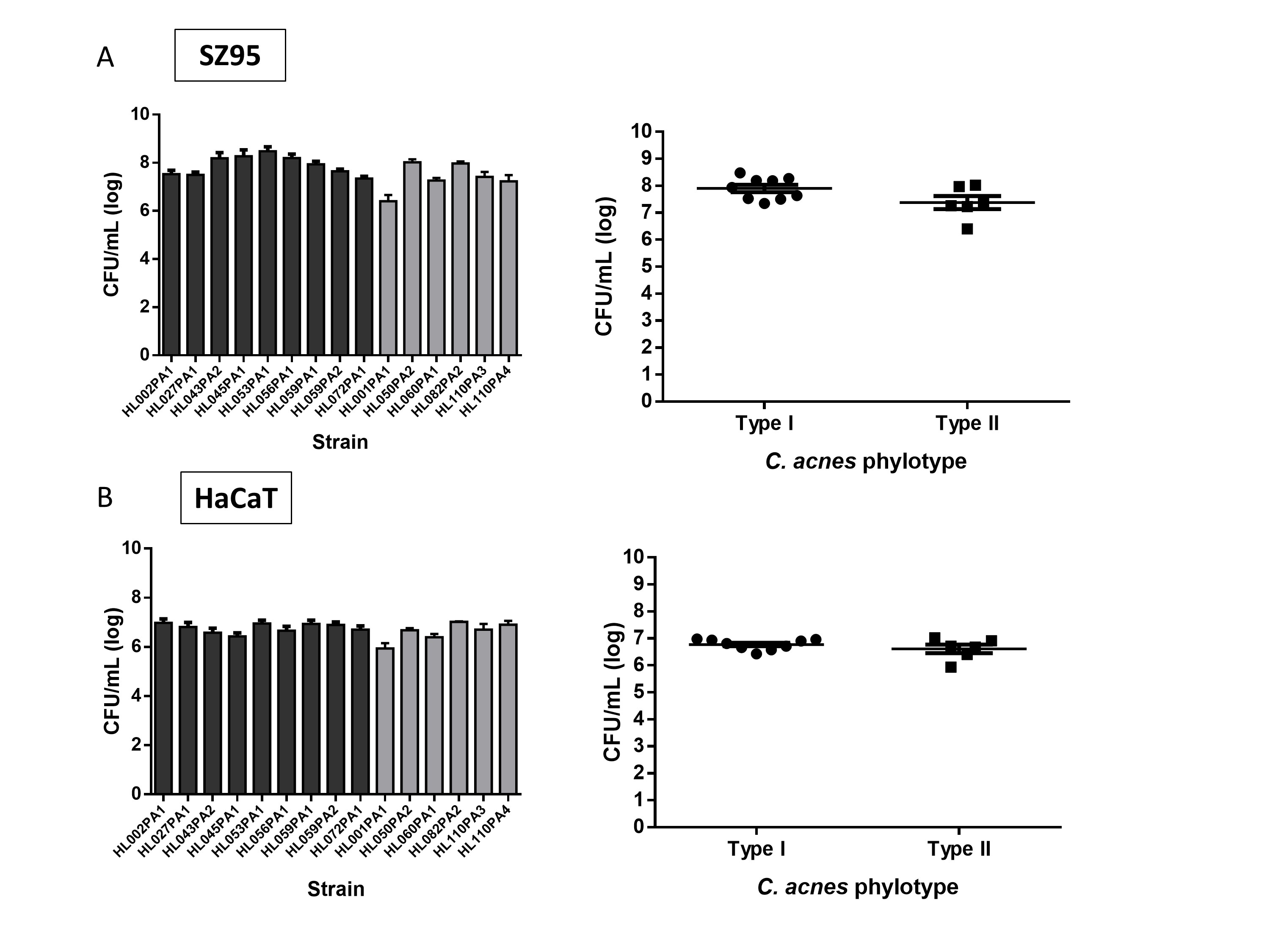

Supplement: Supplementary Data Sheet 2 — C. acnes counts of the non-associated fraction after 48 h infection with SZ95 (A) or HaCaT cells (B). Data shown are mean from at least three biological replicates, error bars indicate SEM. [file Image_2.jpeg]

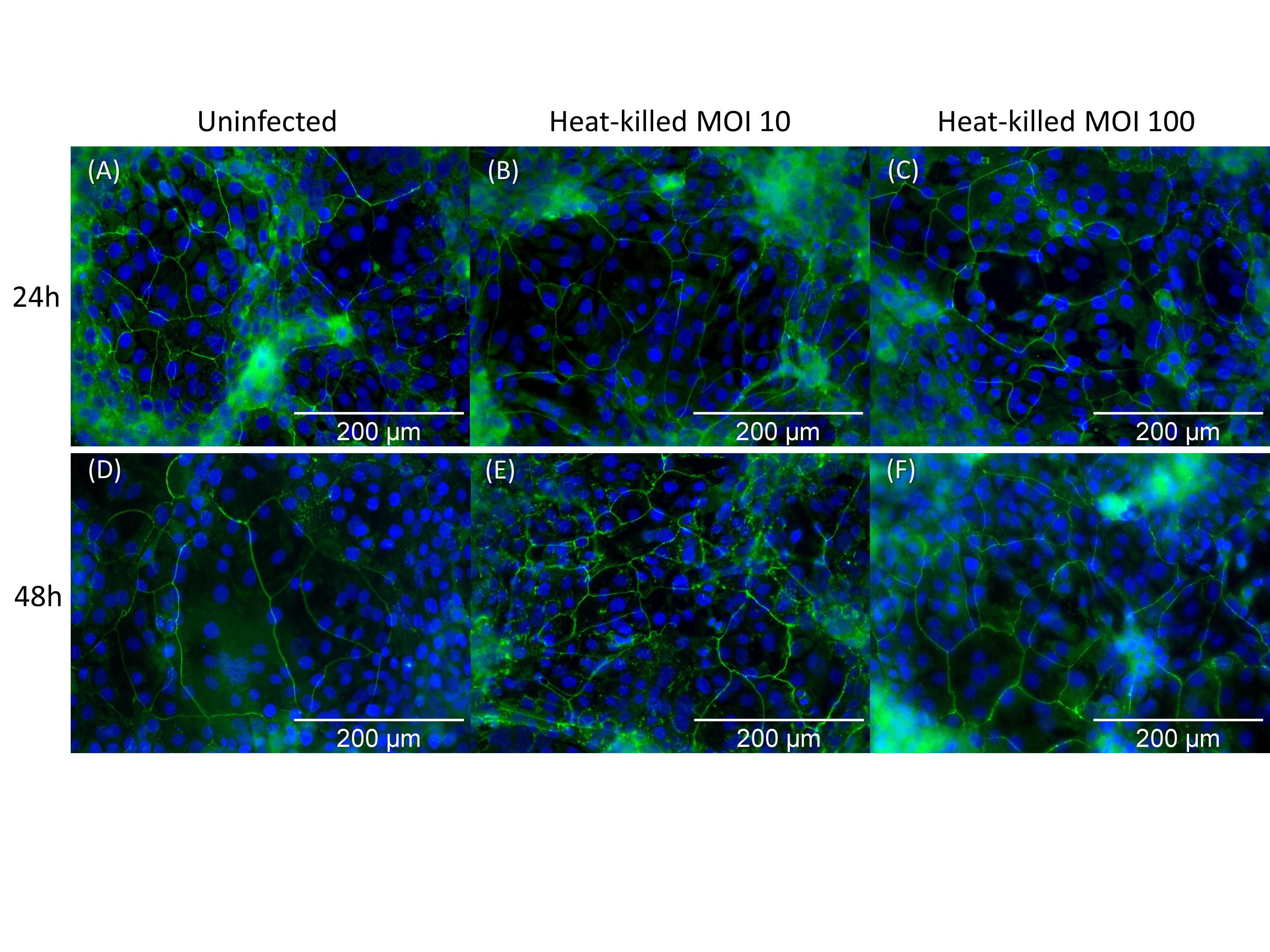

Supplement: Supplementary Data Sheet 3 — Immunocytochemistry staining of the ZO-1 tight junction protein of HaCaT keratinocytes followed by fluorescence microscopy after exposure to heat-killed C. acnes for 24 and 48 h. ZO-1 tight junction proteins and the cell nuclei (DAPI) are visualized in green and blue respectively. Pictures (A–C) show ZO-1 after 24 h exposure to heat-killed HL053PA1: (A) control, (B) exposed to heat-killed C. acnes approximating an MOI of 10:1 for 24 h, and (C) exposed to heat-killed C. acnes approximating an MOI of 100:1 for 24 h. Pictures (D–F) show the still intact ZO-1 protein after 48 h exposure to heat-killed C. acnes: (D) control, (E) heat-killed C. acnes approximating an MOI of 10:1, and (F) heat-killed C. acnes approximating an MOI of 100:1. Total magnification: 599x. Scale bars: 200 µm. [file Image_3.jpeg]

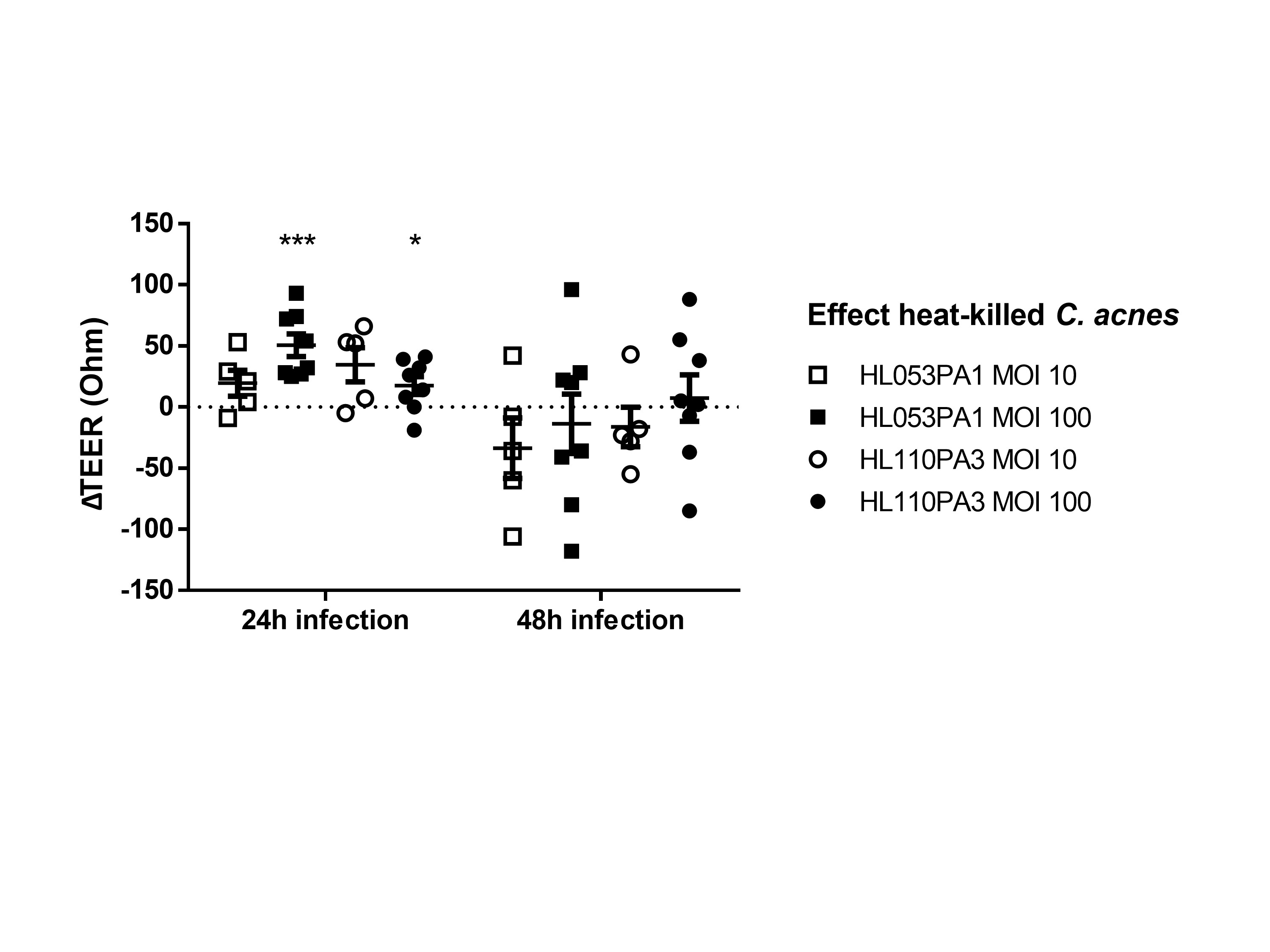

Supplement: Supplementary Data Sheet 4 — The effect of heat-killed C. acnes on the cell lining integrity as measured by TEER. After 24 h exposure a significant increase can be observed at the highest concentrations of heat-killed bacteria. Data represent mean, error bars indicate SEM. * p<0.05, *** p<0.005. [file Image_4.jpeg]
